# Genetic Features of Young and Aged Animals After Peripheral Nerve Injury: Implications for Diminished Regeneration Capacity

**DOI:** 10.1007/s10571-023-01431-8

**Published:** 2023-11-03

**Authors:** Weixiao Huang, Sheng Yi, Lili Zhao

**Affiliations:** 1 Key Laboratory of Neuroregeneration of Jiangsu and Ministry of Education, Co-Innovation Center of Neuroregeneration, NMPA Key Laboratory for Research and Evaluation of Tissue Engineering Technology Products, Nantong University, Nantong, Jiangsu China; 2 School of Medicine & Holistic Integrative Medicine, Nanjing University of Chinese Medicine, Nanjing, China

**Keywords:** Peripheral nerve injury, Aging, Genetic changes, Immune response, Cell cycle

## Abstract

The spontaneous regeneration capacity of peripheral nerves is fundamentally reduced with advancing age, leading to severe and long-term functional loss. The cellular and molecular basis underlying incomplete and delayed recovery of aging peripheral nerves is still murky. Here, we collected sciatic nerves of aged rats at 1d, 4d, and 7d after nerve injury, systematically analyzed the transcriptional changes of injured sciatic nerves, and examined the differences of injury responses between aged rats and young rats. RNA sequencing revealed that sciatic nerves of aged and young rats exhibit distinctive expression patterns after nerve injury. Acute and vigorous immune responses, including motivated B cell receptor signaling pathway, occurred in injured sciatic nerves of both aged and young rats. Different from young rats, aged rats have more CD8^+^ T cells and B cells in normal state and the elevation of M2 macrophages seemed to be more robust in sciatic nerves, especially at later time points after nerve injury. Young rats, on the other hand, showed strong and early up-regulation of cell cycle-related genes. These identified unique transcriptional signatures of aged and young rats help the understanding of aged-associated injury responses in the wound microenvironments and provide essential basis for the treatment of regeneration deficits in aged population.

## Introduction

Peripheral nerves, unlike central nerves, obtain certain regeneration ability after nerve injury and are capable to achieve complete functional recovery (He and Jin 2016). However, with advancing age, the morphologic and functional characteristics of peripheral nerves are largely altered and the regeneration ability of peripheral nerves is fundamentally attenuated (Verdú et al. 2000; Vaughan 1992). Reduced functional recovery of injured peripheral nerves in aged individuals may induce prolonged denervation and progressive atrophy, leading to impaired autonomic, sensory and motor functions (Maita et al. 2023). The aged tendency of population and more importantly, the high morbidity of peripheral neuropathy in aged population, put requirements on the understanding of molecular mechanisms underlying age-related regeneration deficiency and the development of effective therapeutic strategies for the treatment of peripheral nerve injury (Cho et al. 2006).

Success peripheral nerve regeneration rely on the intrinsic regeneration ability of neurons and the microenvironment at lesion sites. The decline of regeneration ability during aging may thus be jointly caused by neurons and cell populations in the wound microenvironment. In the injured peripheral nerves of aged animals, retarded Wallerian degeneration and subsequently delayed clearance of myelin and axonal debris impede axon regeneration (Kang and Lichtman 2013). Transcriptional profiling of sciatic nerves and dorsal root ganglia (DRG) of aged and young mice in naïve conditions and 4d after nerve crush injury showed that Schwann cells in aged sciatic nerves exhibit an attenuated injury response and fail to acquire a dedifferentiated repair phenotype while the intrinsic growth capacity of neurons in aged DRG is not impaired as compared with young animals (Painter et al. 2014). A recent study, however, by comparing transcriptional changes in DRG of aged and young mice at 1d after peripheral nerve injury, demonstrated that neurons process aging-dependent regenerative decline (Zhou et al. 2022). These findings indicate that acute genetic changes after nerve injury, for instance, 1d after nerve injury, may have significant biological implications. Therefore, time series analysis of molecular changes in injured peripheral nerves of aged and young animals, especially analysis of changes at early time points after peripheral nerve injury, is of great significance.

Our laboratory has performed a systematic analysis of sciatic nerves of young rats at multiple time points following nerve crush injury and explored the dynamic involvement of a series of essential biological factors (Yi et al. 2015; Zhang et al. 2019, 2020). In this study, we performed sciatic nerve crush injury to 24-month old rats and collected sciatic nerves at three time points, that is 1d, 4d, and 7d after nerve injury or sham surgery, aiming to obtain gene abundances at the acute phase following injury and to access a more comprehensive understanding of the cellular and molecular changes in the peripheral nerves of aged animals. Differentially expressed protein coding genes were screened and implicated biological processes and signaling pathways were uncovered. Moreover, molecular changes in aged rats were compared with 8-week old young adult rats at equal time points to decipher age-specific alternations and identify genetic causes in the wound microenvironment of regeneration failure in aged individuals.

## Materials and Methods

### Animal Surgery

A total of 27 aged (10–12 months) and 12 young (8–10 weeks) male Sprague–Dawley (SD) rats were purchased from the Experimental Animal Center of Nantong University. Rats were divided to 3 groups (1d, 4d, and 7d) with 6 rats in each group and subjected to sciatic nerve crush injury. Briefly, after anesthetization, sciatic nerve of each rat was exposed by a small incision. The left sciatic nerve at 10 mm above the bifurcation into the tibial and common fibular nerves was crushed with forceps for 3 times with 10 s each time and collected at 1d, 4d, and 7d after surgery. Sham-operated contralateral nerves were served as sham controls. Animal experiments were performed following the Standard Operating Procedures for Laboratory Animal Center of Nantong University and ethically approved by the Animal Experimental Ethical Inspection (No. IACUC20210309-006).

### RNA Extraction and RNA Sequencing

Total RNA was extracted from rat sciatic nerve tissues using TRizol reagent kit (Invitrogen, Carlsbad, CA, USA) and subjected to quality check using an Agilent 2100 Bioanalyzer (Agilent Technologies, Palo Alto, CA, USA). mRNA was enriched by Oligo(dT) beads, fragmented, and reverse transcripted into cDNA. cDNA fragments were purified using QiaQuick PCR extraction kit (Qiagen, Venlo, The Netherlands) and subjected to end repair, poly(A) addition, and ligation. Sequencing was performed in triplicate on the Illumina Hiseq™ 2500/4000 by Gene Denovo Biotechnology Co., Ltd (Guangzhou, China). Sequencing data were deposited in National Center for Biotechnology Information (NCBI) database (BioProject ID: PRJNA691675).

### Bioinformatic Analysis

Reads obtained from sequencing were filtered to remove adapter and reads with low quality bases, aligned with reference genome, and subjected to gene abundance quantification using StringTie software according to the fragment per kilobase of transcript per million mapped reads (FPKM) formula. Gene abundance in nerve injured rats was compared with sham controls to screen differentially expressed genes using DESeq2 software. Gene with an absolute fold change ≥ 2 and a false discovery rate below 0.05 was designated as differentially expressed.

Obtained sequencing data was jointly analyzed with previous sequencing data of young adult rats (deposited in database with the BioProject ID PRJNA394957). Data were subjected to principal component analysis (PCA) using R package gmodels (http://www.r-project.org/) to determine sample relationship. Differentially expressed coding genes were mapped to Gene ontology (GO) terms in the Gene Ontology database (http://www.geneontology.org/) and Kyoto Enrichment of Genes and Genomes (KEGG) pathways to classify gene functions. Differentially expressed coding genes were analyzed using weighted gene correlation network analysis (WGCNA) to separate gene functional networks and analyzed using Ingenuity Pathway Analysis (IPA) software to identify and connect transcription factors with their target genes. The enumeration of immune cells in the wound microenvironment was analyzed using cibersort R software (Newman et al. 2015).

### Immunofluorescence Staining

Sciatic nerves from both young and aged rats before or after crush at 1d, 4d, 7d were dissected. 4% paraformaldehyde was used for post-fixed with 12 h at 4 °C. Then the samples were sliced at thickness of 14 µm after dehydration with sucrose. Before incubate primary antibodies, the sliced should be blocking with Immunofluorescence Blocking Buffer (Beyotime, Shanghai, China) for 1 h at approximately 22 °C. The conditions of primary antibody of IGM (Immunoway, Plano, USA, Cat# YT5774) were 1:100 dilution overnight at 4 °C. After washing with phosphate-buffered saline the slices were then incubated with second antibody Cy3 (1:500; Proteintech, Rosemont, IL, USA Cat# SA00009-2). Samples were then washed three for 10 min each with phosphate-buffered saline, followed by nucleus staining with DAPI (Beyotime). Images were acquired by microscopy (Zeiss, Axio Imager M2m, Carl Zeiss, Oberkochen, Germany) and the intensity of fluorescence were quantified by photoshop software.

## Results

### Identification of Diverse Genetic Changes in Aged and Young Animals After Peripheral Nerve Injury

**Fig. 1:**
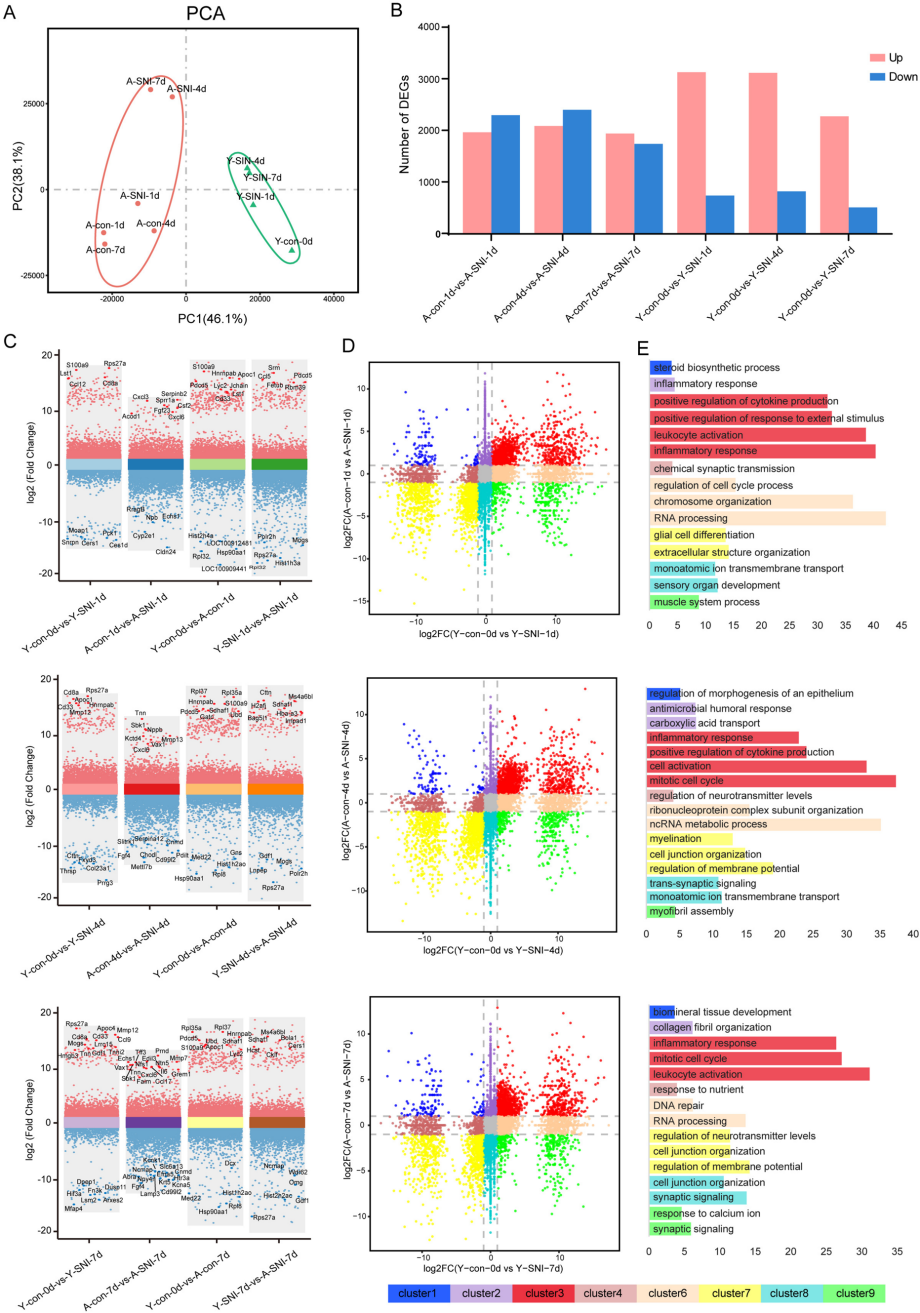
Aged and young animals exhibit unique gene expression patterns post injury. **A** Principal component analysis (PCA) of young and aged samples. Red dots represent aged animal samples from sham groups (A-con-1d, A-con-4d, A-con-7d) and sciatic nerve injured groups (A-SNI-1d, A-SNI-4d, A-SNI-7d), while green dots represent young animal samples from sham groups (Y-con-0d) and sciatic nerve injured groups (Y-SNI-1d, Y-SNI-4d, Y-SNI-7d). **B** The number of differentially expressed coding genes (DEGs) between control and injury group in young or aged animals. **C** Volcano Plot of differentially expressed coding genes in different comparison groups (upper: comparison groups in 1d, middle: comparison groups in 4d, bottom: comparison groups in 7d). Y-axis means log_2_(fold change). Pink dots represent up-regulated genes and blue dots represent down-regulated genes. **D** Scatter plots of young or aged preference differentially expressed coding genes after nerve injury. Eight clusters with distinct colors were divided based on the cutoff of fold-change (2, grey dotted lines). upper: comparison groups in 1d, middle: comparison groups in 4d, bottom: comparison groups in 7d. **E** Importantly enriched GO terms in each cluster in D were shown in different colors. upper: comparison groups in 1d, middle: comparison groups in 4d, bottom: comparison groups in 7d

To explore the genetic mechanisms underlying the age-associated diminished regeneration capacity, gene expression profiles in sciatic nerves of aged rats were subjected to dimension reduction using principal component analysis (PCA). The results demonstrated that genes in sciatic nerves of aged rats underwent sham surgery at 1d (A-con-1d), 4d (A-con-4d), and 7d (A-con-4d) gathered together and were clearly separated from aged rats underwent nerve crush injury (A-SNI-1d, A-SNI-4d, A-SNI-7d). The comparison of gene profiles of injured aged rats showed that there seemed to be a gap between gene expressions of aged rats at 1d after nerve injury and gene expressions of aged rats at relative longer time points. Gene expression profiles in aged rats at 4d and 7d after nerve crush injury were far less distinguishing (Fig. 1A). The cooperative display of gene expression profiles of young rats showed that young animals exhibited different expression patterns as aged animals. The long distance between gene expression profiles of uninjured sciatic nerves of aged rats and young rats suggested huge differences between aged and young animals in naïve conditions (Y-con-0d). Still, in young rats, gene expression patterns were similar at longer time points after nerve injury but different from gene expressions at early time points after injury (Y-SNI-1d, Y-SNI-4d, Y-SNI-7d) (Fig. 1A).

Given that coding genes are directly related to biological functions, the numbers of differentially expressed coding genes in aged and young rats were summarized. Compared with the corresponding uninjured controls, approximately a total of 5000 genes were differentially expressed in aged rats at 1d and 4d after injury, with approximately 2000 genes up-regulated and 3000 genes down-regulated. A moderately smaller number of genes were differentially expressed, especially down-regulated, in aged rats at 7d after injury. Distinctly different from aged rats, the majority of differentially expressed coding genes in young rats were up-regulated and only a small portion of genes were down-regulated (Fig. 1B). The up and down regulated genes in different comparison groups were displayed in volcano plots in detail (Fig. 1C). Moreover, gene profiles of injured aged rats were contrasted with those of young rats to obtain aged preference differentially expressed coding genes after nerve injury (Fig. 1D). Enrichment of these aged preference differentially expressed coding genes to GO terms indicated that at 1d after nerve injury, genes up-regulated in aged but not young rats (cluster 1 and 2) were mostly enriched in terms related with steroid biosynthetic process (cluster 1) and inflammatory response (cluster 2); genes have similar tendency in aged and young rats (cluster 3 and 7) were mainly associated with inflammatory response, leukocyte activation, positive regulation of response to external stimulus (cluster 3) and glia cell differentiation and extracellular structure organization (cluster 7); genes down-regulated in aged but not young rats (cluster 8 and 9) were mostly enriched in terms related with sensory organ development (cluster 8) and muscle system process (cluster 9) (Fig. 1E).

Inflammatory response and mitotic cell cycle were still enriched in both aged and young up-regulated genes at 4d and 7d after nerve injury and cell junction organization and regulation of membrane potential were enriched in both aged and young down-regulated genes at 4d and 7d after nerve injury. Some other GO terms, such as synaptic signaling and ion transport were emerged as aged down-regulated biological processes at 4d and 7d after nerve injury (Fig. 1E).

**Fig. 2:**
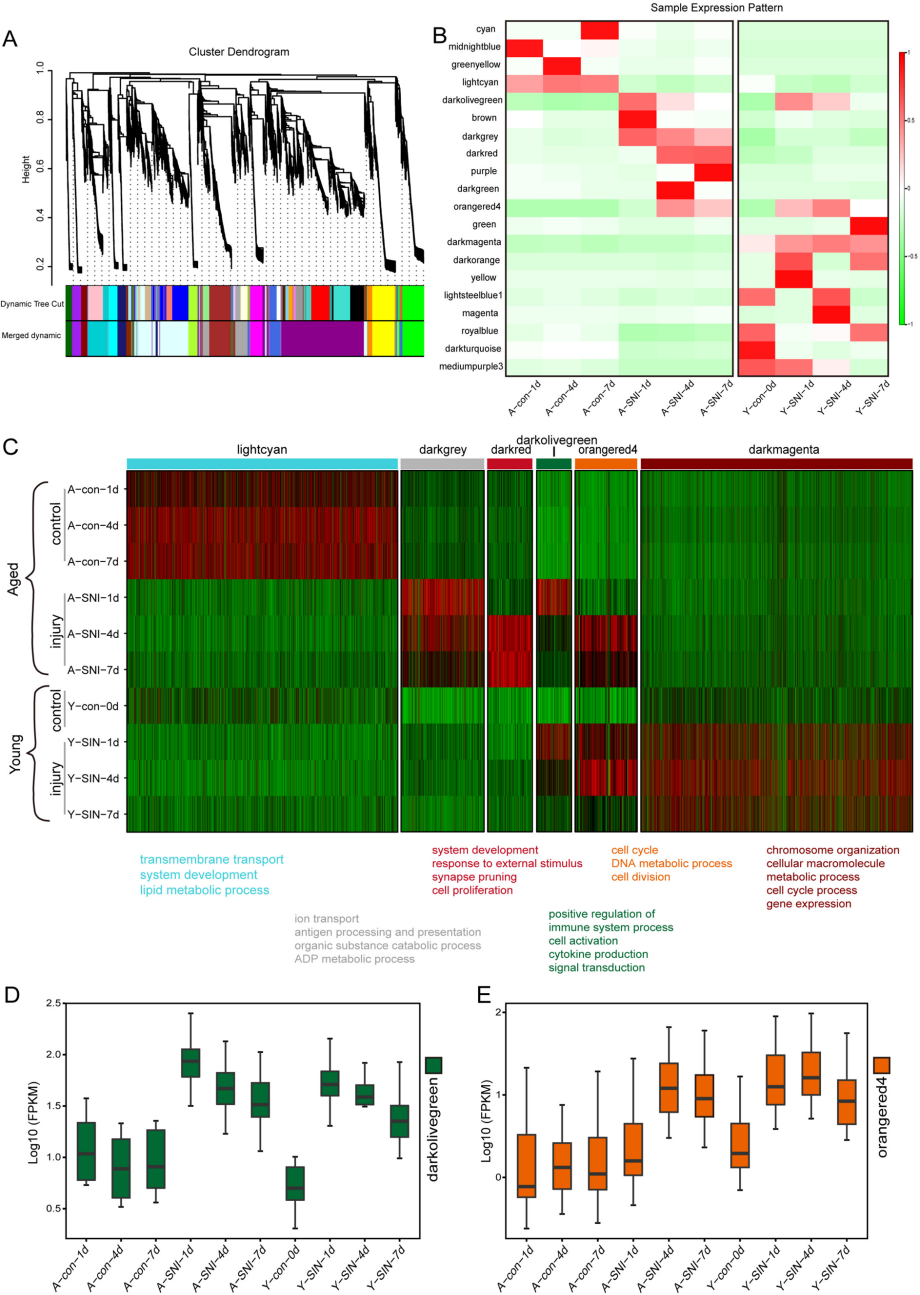
Weighted gene correlation network analysis (WGCNA) analysis reveals differential regulation patterns in aged and young post injury. **A** WGCNA clustering dendrogram. Co-expressed genes were clustered in the same module and labeled with different colors. **B** The correlation between modules and groups. **C** Heatmap showing relative expression of genes in lightcyan, darkgrey, darkred, darkolivergreen, orangedred4, and darkmagenta modules identified by WGCNA of transcriptomes in young and aged animal after nerve injury. Representative Gene ontology (GO) terms of each significantly-regulated module are listed below. **D**, **E** Boxplots showing expression patterns (scaled log2TPM) of hub gene (top 10) in darkolivegreen (**D**) and orangered4 (**E**) module

Regulation patterns of aged and young rats were further investigated using WGCNA analysis. WGCNA analysis elucidated a total of 20 closely co-expressed gene clusters (modules) (Fig. 2A). Uninjured and injured sciatic nerves of aged and young rats showed diverse WGCNA modules. Lightcyan module was specifically highly involved in the intact sciatic nerves of aged rats instead of injured nerves of aged rats or young rats. Darkgray module, on the contrast, was involved in the injured sciatic nerves of aged rats instead of uninjured nerves of aged rats or young rats. Darkred module was characteristically involved in sciatic nerves of aged rats at 4d and 7d after injury. Besides these specific modules, some modules are commonly enriched in both aged and young rats. Genes in both aged and young rats at 1d and 4d after nerve injury were enriched in darkolivegreen module. Genes in aged rats at 4d and 7d after nerve injury as well as young rats at 1d and 4d after nerve injury were enriched in orangered4 module. Young rats displayed some unique features. For instance, compared with aged rats, genes in both uninjured and injured young rats, especially injured young rats, were enriched in darkmagenta module (Fig. 2B).

Genes in these critical WGCNA modules were demonstrated in a heatmap and functionally explored. Genes belonging to lightcyan module, mainly genes in the uninjured aged sciatic nerves, were associated with ion transport, system development, and lipid metabolic process. Genes belonging to darkgrey module, that is chiefly genes in the injured aged sciatic nerves, were associated with ion transport, antigen processing and presentation, organic substance, and catabolic process. Highly related biological processes in other WGCNA modules were discovered as well, including system development, response to external stimulus, and synapse pruning in darkgrey module, positive regulation of immune system process, cell activation, and cytokine production in darkolivegreen module, cell cycle, DNA metabolic process, and cell division in orangered4 module, and chromosome organization, cell macromolecule metabolic process, and cell cycle process in darkmagenta module (Fig. 2C). Darkolivegreen and orangered4 modules attracted most attention as gene belongs to these two modules were robustly differentially expressed after nerve injury for both aged and young rats and showed temporal specificity. Additional exhibition of top 10 genes in darkolivegreen and orangered4 modules showed that consistent with all genes in these two modules, hub genes were expressed at higher levels after nerve injury in both aged and young rats (Fig. 2D, E).

### Identification of Robust Immune Responses in Aged and Young Animals After Peripheral Nerve Injury

**Fig. 3:**
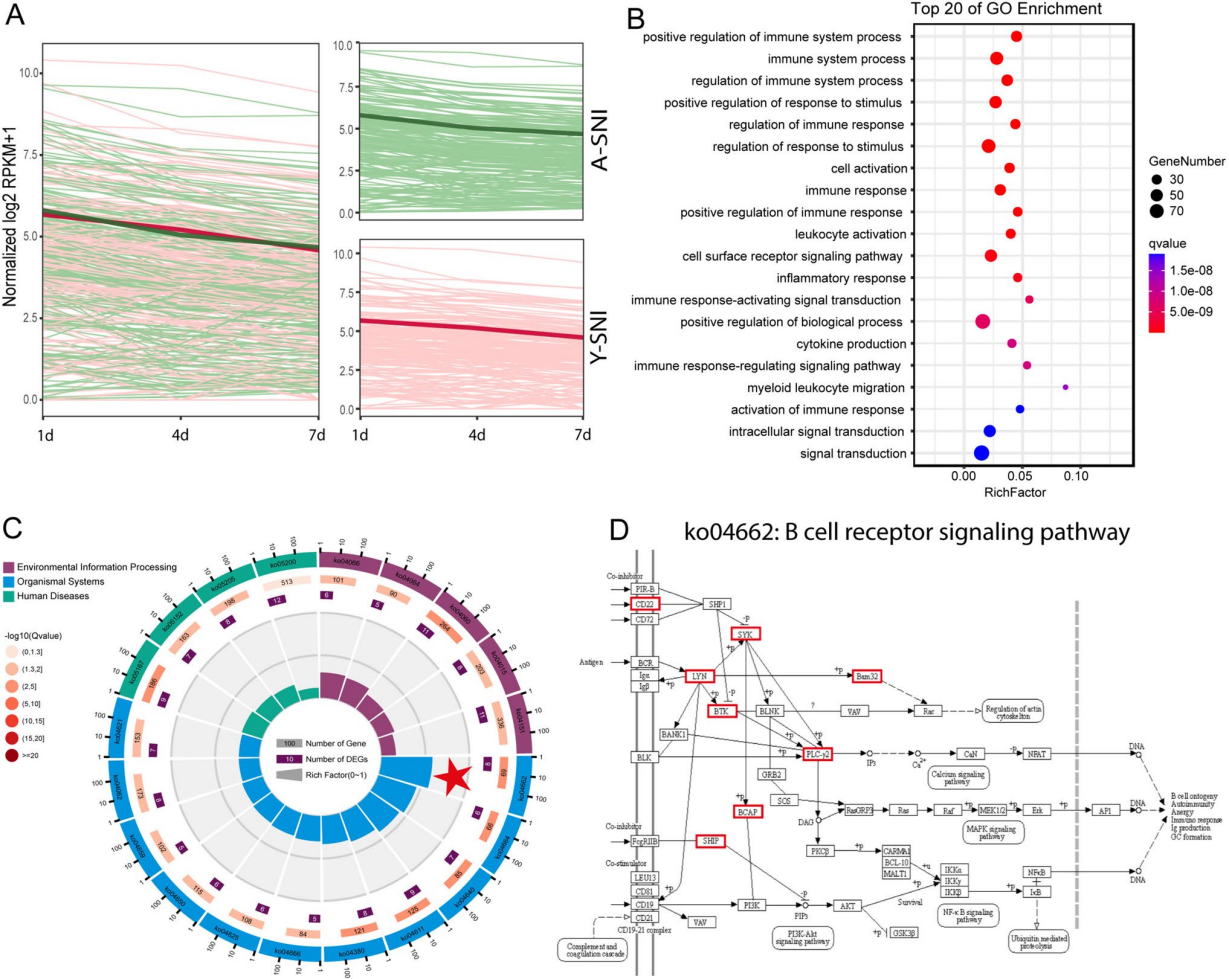
Aged and young animals have different immune cell responses post injury. **A** Fold changes of genes in darkolivegreen module. Red lines represent fold changes of genes in young animals and green lines represent fold changes of genes in aged animals. **B**, **C**: The enriched GO terms (**B**) and Kyoto Enrichment of Genes and Genomes (KEGG) pathways (**C**) of genes in darkolivegreen module. The most significant KEGG pathway was labeled with red star. **D** The schematic network of B cell receptor signaling pathway (Ko04662). Up-regulated genes after injury were marked with red box. Signaling pathway diagram was adopted from KEGG database

A full demonstration of genes in WGCNA darkolivegreen module indicated that as compared with their corresponding uninjured control, in both aged and young rats, the expression levels of genes in darkolivegreen module increased to a log_2_RPKM + 1 value of approximately 6 at 1d after nerve injury. The expressions of the majority of genes in darkolivegreen module were slightly decreased at 4d and 7d, especially in young rats. Still, the expressions of these genes remained at high levels as compared with the uninjured controls (Fig. 3A). GO analysis showed that genes in darkolivegreen module were primarily associated with immune responses and immune response-regulated activities (Fig. 3B). The substantial involvement of immune responses was identified by KEGG analysis as well, with B cell receptor signaling pathway (Ko04662) being the most significant KEGG pathway (Fig. 3C). Many genes that participate in the antigen-receptor signals of B cells, including CD22 (CD22 Molecule), LYN (LYN proto-oncogene, Src family tyrosine kinase), BTK (Bruton agammaglobulinemia tyrosine kinase), SYK (Spleen Associated Tyrosine Kinase), Bam32 (dual adaptor for phosphotyrosine and 3-phosphoinositides 1), PLC-γ2 (phospholipase C, gamma 2), SHIP (also called *INPP5D*, Inositol Polyphosphate-5-Phosphatase D), and BCAP (also called *ODF2L*, Outer Dense Fiber Of Sperm Tails 2 Like), were elevated in both aged and young rats after nerve injury (Fig. 3D).

**Fig. 4:**
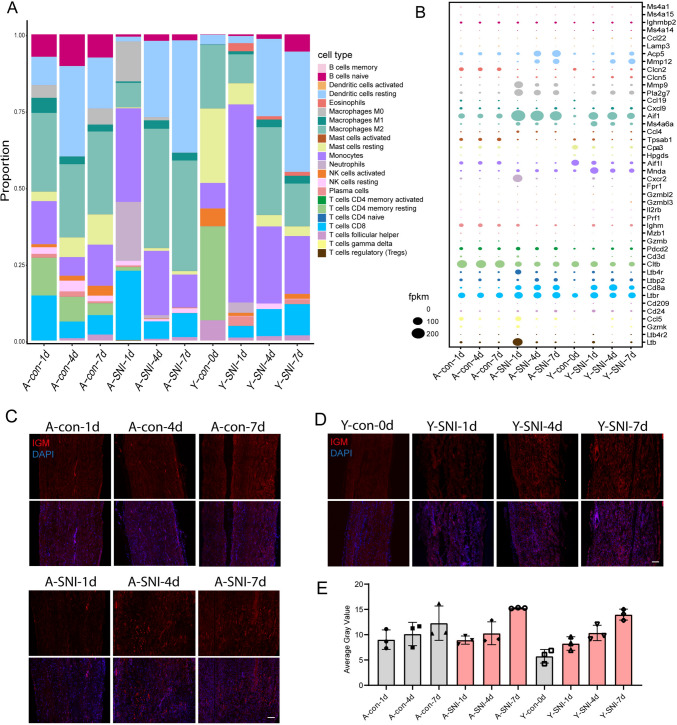
The immunoinfiltration of aged and young rats after sciatic nerve injury. **A** The proportion of different immune cells in different groups. **B** The expressions of markers of different types of immune cells in different groups. **C** The immunostaining of immunoglobulin heavy chain (IGM) in sciatic nerve of aged rat before or after injury. Red represents IGM and blue represents DAPI (cell nucleus), the scale bar = 100 μm. **D** The immunostaining of immunoglobulin heavy chain (IGM) in sciatic nerve of young rat before or after injury. Red represents IGM and blue represents DAPI (cell nucleus), the scale bar = 100 μm. **E**: IGM signal intensity statistics, Y axis represents the average gray value of IGM signal, X axis represents different groups, *n* = 3

Given that immune responses are cooperatively regulated by various types of immune cells, we compared immunoinfiltration through the proportion of different immune cells in each group (Fig. 4A). In general, immune cells exhibited dissimilar distributions even in the intact sciatic nerves of aged and young rats. Firstly, there was a significant proportion of CD8 + T cells in aged rats both in shamed and injury groups while the proportion of CD8 + T cells in control group in young rats was very low. The expression of Cd8 was very low in uninjured young rats when compares with aged animals (Fig. 4A and B). This phenomenon was consisted with the report before in DRGs of aged rats (Zhou et al. 2022), indicated the chronic inflammation in aged animal. The proportion of memory resting T cells in the uninjured young sciatic nerve was obviously much larger while the proportion of B cells was noticeably much smaller as compared with the uninjured sciatic nerves of aged rats (Fig. 4A and B). To conform these results, we explored the expression of IGM, a marker of B cells, through immunostaining in the sciatic nerve of both old and young rats before and after injury. We found that in the absence of injury, the sciatic nerve of old rats expressed more IGM than that of young rats, but after injury, the IGM of both old and young rats continued to increase (Fig. 4D, E), which just consistent with immune infiltration analysis.

Following sciatic nerve injury, larger amounts of dendritic cells and macrophages were observed in both aged and young rats, with increased macrophages occurred at early time points after nerve injury and increased activated dendritic cells occurred at later time points after nerve injury (Fig. 4A). Aged and young rats also displayed specific immune responses to nerve injury. In aged rats, increased proportion of neutrophils and elevated expression of its marker Cxcr2 (C-X-C Motif Chemokine Receptor 2) as well as ip-ncreased proportion of regulatory T cells (Tregs) and elevated expression of its maker Ltb (Lymphotoxin Beta) were observed at 1d after nerve injury. Higher level of Acp5 (Acid Phosphatase 5, Tartrate Resistant) and larger amount of resting dendritic cells was identified in aged rats at 4d and 7d after nerve injury. Compared with aged rats, in young rats, increased proportion of Monocytes and elevated expression of its marker Mnda (Myeloid Cell Nuclear Differentiation Antigen) at 1d after nerve injury (Fig. 4A and B). These findings demonstrated that aged and young rats underwent robust but unique immune responses.

### Identification of Activated Cell Cycle in Young Animals After Peripheral Nerve Injury

**Fig. 5:**
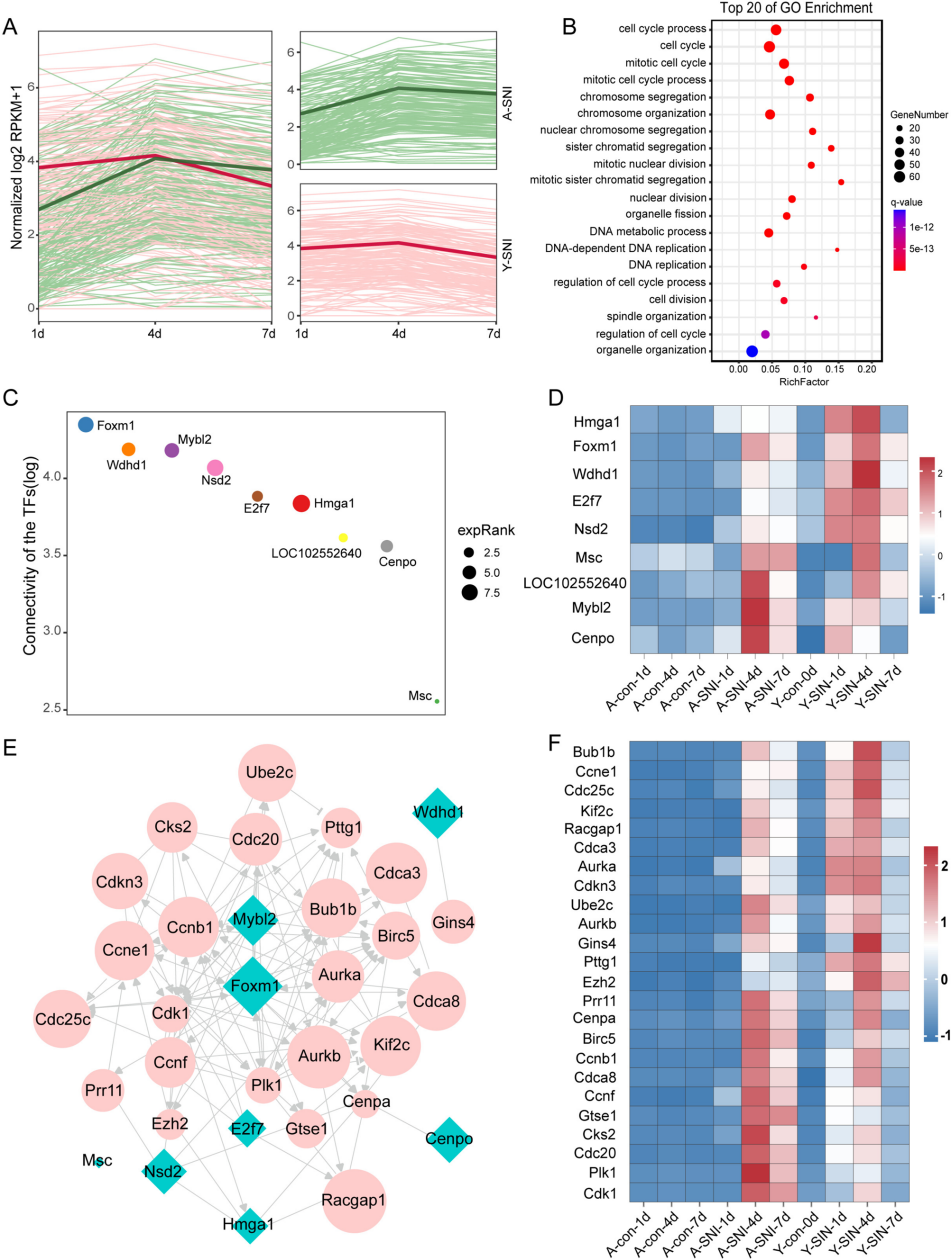
Cell cycle is more active in young animal post injury. **A** Fold change of genes in orangered4 module. Red lines represent fold change of genes in young animals and green lines represent fold change of genes in aged animals. **B** The enriched GO terms of genes in orangered4 module. **C** The ranking of transcription factors based on the connectivity within the orangered4 module. The size of plot represents gene expression at 1d after injury. **D** The expressions of transcription factors within orangered4 module, the grey lines indicated interaction between transcription factors and targets. **E** The interactions between transcription factors and targets within orangered4 module. The transcription factors were labeled with cyan diamond and target were labeled with pink circle. The grey lines indicated interaction between genes which were predicted from IPA software. **F** The expressions of the targets of these transcription factors within orangered4 module

The dynamic expression patterns of genes in orangered4 module, another WGCNA module significantly involved nerve injury-mediated expression changes, were displayed in details as well. For both aged and young rats, genes in orangered4 module increased to a similar level at 4d after nerve injury. However, compared with aged rats, in young rats the immediate injury response of genes in orangered4 module was more robust and genes increased to a higher level at 1d after nerve injury (Fig. 5A). These differentially expressed genes were closely associated with cell cycle, chromosome segregation, nuclear division, DNA metabolism, DNA replication, and cell division (Fig. 5B).

To explore the switch of these cell cycle related genes, the connectivity and expressions of transcription factors were investigated. There are 9 transcription factors in orangered4 module, with Foxm1 (Forkhead Box M1) have highest connectivity and Msc (Musculin) have lowest connectivity (Fig. 5C). In young rats, most transcription factors and target genes were rapidly up-regulated in 1d after nerve injury while in aged rats, transcription factors and target genes showed increased trends at a later time point, that is 4d after nerve injury. Among these differentially expressed transcription factor, Hmga1 (High Mobility Group AT-Hook 1), Foxm1, Wdhd1 (WD Repeat And HMG-Box DNA Binding Protein 1), E2f7 (E2F Transcription Factor 7) and Nsd2 (Nuclear Receptor Binding SET Domain Protein 2) were first increased and shown higher expression in young rats after nerve injury while LOC102552640, Mybl2 (MYB Proto-Oncogene Like 2) and Cenpo (Centromere Protein O) have higher expression at 4d after injury in aged rats (Fig. 5D).

The targets of these transcription factors and the relationship between them were further explored. Foxm1 appears as the hub of the network, and Ccnb1 (Cyclin B1) also shown higher connectivity among the targets (Fig. 5E). The expression of these targets shown similar to the transcription factors that most of them were rapidly up-regulated in 1d in young but not in aged rats. Among the targets, Pttg1 (PTTG1 Regulator of Sister Chromatid Separation, Securin) and Ezh2 (Enhancer of Zeste 2 Polycomb Repressive Complex 2 Subunit) have little up-regulated after injury in aged rats, but shown obvious higher expression after injury in young rats. Although the expression of Bub1b (BUB1 Mitotic Checkpoint Serine/Threonine Kinase B), Ccne1(Cyclin E1), Cdc25c (Cell Division Cycle 25C) and Gins4 (GINS Complex Subunit 4) were both increased after injury in young and aged rats, they appear higher expression in young animal. In addition, some genes like Cks2 (CDC28 Protein Kinase Regulatory Subunit 2) and Plk1 (Polo Like Kinase 1) shown little change at 1d but rose rapidly at 4d after injury in aged rats compared with young rats (Fig. 4D).

## Discussion

Sequencing analysis offers accurate measurement of transcript expressions and therefore depicts transcriptional characteristics of tissues and organs under numerous physiological and pathological conditions, including peripheral nerve repair and regeneration process. RNA sequencing performed on DRG from aged and young mice discloses powerful changes in immune-related gene expression in aged animals at 1d after sciatic nerve injury and reveals the mechanisms underlying age-associated regenerative decline from the aspect of the interaction between neurons and immune cells (Zhou et al. 2022). Herein, instead of DRG, we focused on nerve segments at the injured sites and compared transcriptional changes in injured sciatic nerves from aged and young animals. Transcriptional profiling of injured sciatic nerves at 1d, 4d, and 7d were explored to obtain a global view of both acute and post-acute injury responses, aiming to decipher age-associated changes in the wound microenvironment.

RNA sequencing and WGNCA analysis of meta-genes discerned distinctive gene expression profiling and functional clusters in the sciatic nerves of aged and young rats after nerve crush injury. Besides the exclusive enrichment of WGCNA lightcyan module in aged rats underwent sham surgery, darkgrey and darkred modules in aged rats underwent sciatic nerve crush injury, and darkmegenta module in young rats underwent sciatic nerve crush injury, two WGCNA modules, i.e., darkolivegreen module and orangered4 module, were highly involved in both aged and young rats after nerve injury and thus were investigated in detail.

Darkolivegreen module, a module of genes closely associated with immune responses, is rapidly activated in injured sciatic nerves regardless of age. Actually, immediately and sustained activated immune responses, such as agranulocyte/granulocyte adhesion and diapedesis, IL-6 signaling, and IL-10 signaling, have been previously identified in the injured sciatic nerves of young animals (Yi et al. 2015; Xing et al. 2017). Here, we find that aged animals also exhibit robust immune responses following nerve injury. The identification of B cell receptor signaling pathway as the most significantly enriched KEGG pathway points to the importance of B cell-based adaptive immunity in the injured microenvironment. Although neonatal B cells may be beneficial for cell proliferation and tissue regeneration, adult B cells are generally associated with impaired functional recovery (Tan et al. 2023; Dowery et al. 2021; Frede et al. 2022; Fleig et al. 2021). Rodents lacking mature B cells and T cells exhibited increased neuron survival, elevated remyelination, and improved recovery after femoral nerve injury (Mehanna et al. 2014). These findings indicate that activated B cell-based adaptive immune responses in wound microenvironment may be harmful for subsequent nerve regeneration. In addition, the characterizations of cellular composition of sciatic nerves of sham injured aged and young rats from their gene expression profiles show that compared with sciatic nerves of young rats, sciatic nerves of aged rats have a larger number of B cell population at homeostatis. Elevated B cells in naïve sciatic nerves of aged rats may be a contributing factor for the moderated regeneration capacity.

Immune cell composition characterization further points out the differences between injured sciatic nerves from aged and young rats and demonstrates the appearance of plentiful M2 macrophages in sciatic nerves of aged rats at 4d and 7d after nerve injury. M2 macrophages represent alternatively activated immunosuppressive cells that create a favorable microenvironment and promote peripheral nerve repair (Chen et al. 2015; Liu et al. 2019). An earlier study determines the expressions of genes coding for anti-inflammatory cytokines in peripheral nerves of aged and young rats and finds that at 1d after nerve injury, the up-regulation of M2 markers is attenuated in older rats (Scheib and Höke 2016b, 2016a). Here, we characterize genetic changes at more time points, examine macrophage responses in sciatic nerves of aged and young rats, and find the increase of M2 macrophages in aged rats at relatively later time points following injury. Our findings are consistent with previously observed persistent elevated expression of cytokines, including M2 macrophage markers IL6, IL10, CXCL13, in aged mice at long time period after sciatic nerve crush injury (Büttner et al. 2018). Whether these emerging M2 macrophages at later time points post nerve injury in aged rats play a compensatory role for tissue remodeling and nerve regeneration remains to be further explored. Currently, manipulating immune cells has been utilized as a powerful strategy for promoting tissue regeneration (Julier et al. 2017; Zarubova et al. 2022). Profiling immune cell compositions in the wound microenvironment may contribute to the understanding of the dynamic changes and biological functions of immune cells and thus may provide important basis for the immune-engineering therapeutic avenue toward the treatment of peripheral nerve injury.

Our bioinformatic study deciphers the differences of immune responses in injured peripheral nerves of rats of different ages and thus emphasizes the essential roles of immune cells during nerve regeneration. Notably, other cell types, for instance Schwann cells, also play important roles in diminished regeneration capacity of peripheral nerves in aged animals. For instance, it has been well demonstrated that c-Jun activation promotes the reprogramming of Schwann cells to a repair phenotype (c-Jun reprograms Schwann cells of injured nerves to generate a repair cell essential for regeneration). Aged animals have reduced c-Jun expressions and genetically restoring c-Jun abundance in Schwann cells is capable of facilitating nerve regeneration (Failures of nerve regeneration caused by aging or chronic denervation are rescued by restoring Schwann cell c-Jun). Besides immune cells and Schwann cells, the changes and biological functions of other cell populations during aging, such as endothelial cells and pericytes, are also worth of investigating.

The enrichment of orangered4 module in aged and young rats with young rats indicates the importance of activated cell cycle-related genes during nerve regeneration. A comparison study of the intact sciatic nerves of 1-week-old and 12-month-old male SD rats reveals that aging induces altered expression patterns of cell viability and cell proliferation-associated genes (Analysis of transcriptome sequencing of sciatic nerves in Sprague–Dawley rats of different ages)(He et al. 2018). Our data implied that besides the different basic levels under the healthy condition, in aged animals, these genes may exhibit more robust changes as compared with in young animals following nerve injury, and thus the regenerative capacity of cells in the wound microenvironment may be further declined. Identification of transcription factor coding genes and their targets in orangered4 module show the essential role of Foxm1. Transcriptional activator Foxm1 is involved in DNA replication and cell proliferation and is frequently highly expressed in tumor, including malignant peripheral nerve sheath tumor (Liao et al. 2018; Borhani and Gartel 2020; Yu et al. 2011). Furthermore, elevated Foxm1 is associated with accelerated the regeneration of numerous tissues and organs, such as liver, renal tubule, and heart (Izumi et al. 2018; Sinha et al. 2020; Wang et al. 2020). Foxm1 is also critical for spinal cord regeneration as reduced number of neurons in the regenerating spinal cord is observed in Foxm1 knockout tadpoles (Pelzer et al. 2021). However, the involvement of Foxm1 in peripheral nerve injury and repair process has not been explored yet. Our bioinformatic analysis results demonstrate the central locate of Foxm1 in regenerating sciatic nerves of both aged and young rats, the functional experiments of it in further may give us more evidence about its status.

Taken together, our current study reveals cellular and molecular changes in the wound microenvironment of aged and young animals and deciphers commonly activated biological processes as well as aging-specific injury responses following peripheral nerve injury. A better understanding of dynamic genetic changes underlying aging-dependent regenerative failure may help to treat incomplete peripheral nerve regeneration in the aging population.

## Data Availability

Sequencing data of sciatic nerves of aged rats subjected to sciatic nerve crush injury or sham surgery at 1d, 4d, and 7d after surgery were deposited in National Center for Biotechnology Information (NCBI) database (BioProject ID: PRJNA394957). Sequencing data of sciatic nerves of the young rat are available in the NCBI database with the accession numbers PRJNA394957.
